# Current Advances in the Use of Mushrooms as Therapeutics for Lung Cancer: A Review

**DOI:** 10.3390/molecules30061322

**Published:** 2025-03-14

**Authors:** Edward Thato Khunoana, Sanah Malomile Nkadimeng

**Affiliations:** Department of Life and Consumer Sciences, College of Agriculture and Environmental Sciences, Florida Campus, University of South Africa, Private Bag X6, Florida 1710, South Africa; khunoet@unisa.ac.za

**Keywords:** lung cancer, mushrooms, antiproliferative, adenocarcinoma, A549, apoptosis

## Abstract

Medicinal mushrooms have become increasingly important in the pharmaceutical industry because they contain a wealth of bioactive compounds and offer various nutritional benefits. These qualities also contribute to their widespread use in cooking. Global mycologists have suggested that a deeper understanding of mushrooms can aid in treating a variety of cancers at different stages. The excellent anticancer potential of fungi has inevitably attracted the attention of researchers, given the ever-increasing number of cases of lung cancer. Thus, the purpose of this review was to compile and synthesize the existing scientific literature about the potential of mushroom extracts particularly towards lung cancer prevention. A comprehensive literature search was conducted in electronic databases to identify relevant studies for the review. We found that 26 distinct kinds of mushrooms, prepared in over 10 different solvents, were used to make extracts that decreased the viability of different types of lung cancer cells (A549, NCI-H460, 3LL, H1264 cells etc.). We also examined a range of experimental models, including cell cultures (in vitro), mouse models (in vivo), as well as case studies and randomized controlled trials. The investigated mushrooms’ effective mechanisms included: a reduction in the growth of cancer cells; an imbalanced percentage of cells in different phases of the cell cycle; an increase in autophagy and phagocytosis; an enhanced immune response; and the induction of cell apoptosis through the upregulation of pro-apoptotic factors and the downregulation of anti-apoptotic genes. Considering that mushrooms are consumed as a daily supplement, their potent pro-apoptotic properties and high antiproliferative efficacy are advantageous and could provide a model for further studies in this field as well as novel drug therapies and cancer treatments.

## 1. Introduction

Cancer creates a substantial burden on healthcare, society, and the economy, with lung cancer (LC) being a major contributor [1]. Lung cancer is widespread and continues to be the primary cause of cancer-related mortality worldwide [2]. In 2020, LC was projected to cause approximately 2.21 million new cancer cases, representing around 11.4% of all reported cancer diagnoses [3]. Furthermore, LC led to an estimated 1.80 million deaths, making up approximately 18% of all cancer-related fatalities [3]. LC is categorized into two main histological types: non-small-cell lung carcinoma (NSCLC) and small-cell lung carcinoma (SCLC). NSCLC is more prevalent, accounting for 80 to 85% of LC cases [4]. Managing and treating LC has always been a difficult and complex task. Cancer treatments are diverse, including surgery, anticancer drug treatment, radiotherapy, and systemic therapy (chemotherapy and targeted biological therapies) [5]. The current anticancer drugs available on the market are expensive and not target specific, leading to the development of drug resistance and even causing several side effects in clinical chemotherapy [6]. Therefore, it is important to develop novel and effective anticancer agents with low toxicity. Mushrooms have been used in traditional medicines globally due to their immunomodulatory, anticancer, antioxidant, and anti-inflammatory activities. Currently, out of the 14,000 different mushroom species, about 700 have been found to exhibit medicinal properties [7]. Medicinal mushrooms not only have nutritional value and pharmacological efficacy but are also economical, easy to grow, and suitable for promotion worldwide. Although there is supporting evidence for the therapeutic effects of mushrooms, further research is necessary to translate these mushroom-based substances into clinical practice [8]. Recent studies have revealed that certain mushrooms possess strong anticancer properties against lung cancer, making them promising complementary therapy for patients. Despite the fact that many mushroom species remain underexplored, researchers are isolating novel bioactive compounds for further investigation. For example, lentinans found in shiitake mushrooms have been linked to enhanced effectiveness in lung cancer treatment [9]. Several reports suggest that consuming mushrooms or mushroom-based extracts is associated with a reduced incidence of cancer and improved survival rates [10,11]. Although there is limited information on the connection between mushroom consumption and lung cancer risk factors, it is important to consider studies indicating potential in reducing lung cancer risk [6]. To date, various studies have investigated the anticancer properties of mushroom extracts and bioactive components; however, no prior research has precisely summarized the findings related to lung cancer. This review is the first comprehensive examination of all eligible findings concerning the anti-lung cancer properties of mushrooms. Consequently, the aim of our research is to examine the existing literature on the topic and to find mushroom extracts and bioactive compounds exhibiting potential anti-lung cancer efficacy. Our objective is to furnish information regarding the prospective therapeutic application of mushrooms in lung cancer treatment and their anticancer mechanisms. This review will assist researchers and clinicians in formulating evidence-based preclinical and clinical studies to evaluate the anticancer efficacy of mushrooms and their active components in lung cancer.

## 2. Materials and Methods

### 2.1. Literature Search Strategy

A comprehensive literature search was conducted to identify relevant studies for the review. The search was carried out in electronic databases, including PubMed, Scopus, and Google Scholar. Keywords used included “mushrooms used for lung cancer”, “biochemicals in lung cancer mushrooms”, “health benefits of lung cancer mushrooms”, “anti-cancer mechanisms of lung cancer mushrooms”, “mushrooms as anti-cancer agents”, “mushroom effects on adenocarcinoma”, and related terms.

### 2.2. Inclusion Criteria

This review included over 58 studies centered on investigating the effects and biological compound composition of mushrooms against lung cancer. It comprised research outlining the various health advantages of mushrooms, including their potential mechanism of action and anti-cancer activities. Research chosen for inclusion came from credible journals and were only included if the data were adequate and pertinent.

### 2.3. Exclusion Criteria

This evaluation excluded studies primarily focused on cancers other than lung cancer, such as those that dealt with breast, liver, and prostate cancers. Non-English articles lacking comprehensive translations were excluded. Studies with inadequate pertinent data were excluded.

## 3. Results

### 3.1. Uses of Mushrooms in Lung Cancer Therapy

**Figure 1:**
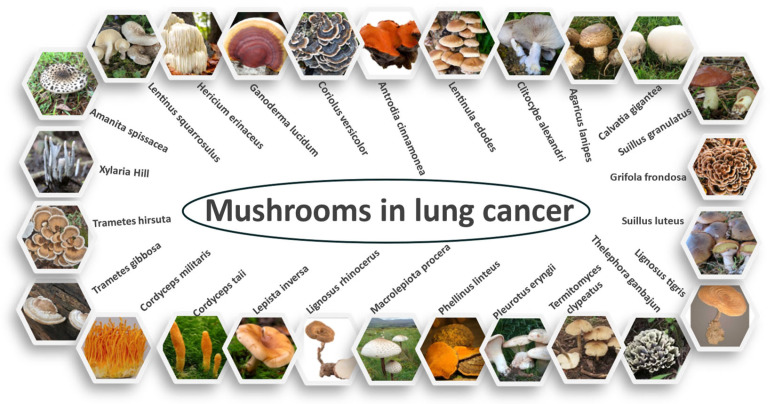
Medicinal mushrooms used to treat lung cancer [12].

A wide range of medicinal mushrooms were identified from various studies. In total, 26 mushrooms, as illustrated in Figure 1, were found to be used to treat lung cancer. The mushrooms originate from various regions and are used traditionally to treat lung cancer. The majority of the mushrooms are predominantly used in Asian traditions. The mechanism and phytochemical composition of the medicinal mushrooms are listed on Table 1.

### 3.2. Agaricaceae

#### 3.2.1. *Agaricus lanipes* 

*Agaricus lanipes* (*A. lanipes*) methanol extracts were assessed for their antiproliferative activity and apoptotic activity against A549 lung cancer cells. in a study by [13]. *A. lanipes* extracts were evaluated for their antiproliferative activity and apoptotic involvement. The results demonstrated that mushroom extract reduced the growth of A549 cells in a dose-dependent manner compared to the untreated control cells. Owing to the patterns that are reliant on both time and dose observed in the viability of A549 cells, the expression changes of genes, including Rel A (p65), Bax, Bcl-2, caspase-3, caspase-9, p21, p53, and Cyclin D1 (CCND1), were assessed at 24-, 48-, and 72-h following treatment of A549 cells with varying concentrations of the extract. Based on these findings, it was proposed that *A. lanipes* extract can inhibit the cell cycle by downregulating CCND1 and causing A549 lung adenocarcinoma cells to undergo apoptosis via modifying the expression of Bax, Bcl-2, and caspases. Given that the mushroom is consumed as a daily supplement, its great antiproliferative efficacy and strong pro-apoptotic effects are beneficial and may serve as a model for future research in this field as well as a source for novel medication formulations and treatments for cancer.

#### 3.2.2. *Calvatia gigantea* 

*Calvatia gigantea* (*C. gigantea*) is a species of edible fungus that is a member of the Lycoperdaceae family. According to research, *C. gigantea* is one of the puffballs. The puffball’s proteins and peptides have been shown to have anti-tumor effect. The anticancer factor calvacin was first isolated from *C. gigantea*, which belongs to the Basidiomycota phylum, and which has also long been used as a medicinal food. It was shown that *C. gigantea* inhibits the growth of human lung cancer cells by downregulating the genes Akt, CDK4, CCND1, and CCND2, which are important for cell cycle arrest in the G1/S phase. Simultaneously, *C. gigantea* extract induces apoptosis by downregulating the expression of the anti-apoptotic protein Bcl-2 and increasing the apoptotic factors Bax, p53, caspase-3, and caspase-9. Finally, *C. gigantea* extract may be a significant agent for treatment of lung cancer as a single agent. In conclusion, *C. gigantea* extract might be a useful treatment for lung cancer. Therefore, more preclinical and clinical research is needed to further understand the greatest effects of *C. gigantea* [17].

#### 3.2.3. *Macrolepiota procera* 

Tumor metabolism has emerged as a new biomarker for cancer, characterized by the heightened expression of enzymes involved in metabolic processes, such as glycolysis and the pentose phosphate pathway (PPP). Enzymes from the PPP, like glucose 6-phosphate dehydrogenase (G6PD) and 6-phosphogluconate dehydrogenase (6PGD), are essential for fulfilling the energy and biosynthetic requirements of cancer cells. Due to their overexpression in various tumors, G6PD and 6PGD are being explored as potential therapeutic targets. A study by [32], examined the ability of ethanol extracts from *Macrolepiota procera* (*M. procera*) to inhibit the activity of G6PD and 6PGD. Additionally, the extracts were tested for their effects on the proliferation of human lung cancer cells (A549), revealing that *M. procera* achieved a 94% inhibition of G6PD and demonstrated cytotoxic effects against A549 cells. Based on the results, it was concluded that *M. procera* inhibited G6PD as part of its anticancer mechanism against A549 lung cancer cells. Therefore, it is crucial to discover and develop G6PD inhibitors from mushrooms, such as *M. procera*, as they may be ideal treatment options for lung cancer. Overall, the study supported the application of this edible mushroom species as a medicinal agent with anti-cancer properties; nevertheless, more in vivo investigation is recommended to confirm *M*. *procera*’s potential against lung cancer [32].

### 3.3. Amanitaceae

#### *Amanita spissacea* 

The methanol (MeOH) extract from the fruiting body of *Amanita spissacea* (*A. spissacea*) showed significant cytotoxic effects against human lung cancer cells in vitro during preliminary testing. The extract was evaluated on four human lung cancer cell lines with different p53 statuses: “A549 cells with wild-type p53, H1264 cells with mutated p53, and H1299 and Calu-6 cells lacking p53”. The MeOH extract consistently reduced cell viability across all cancer cell lines in a dose-dependent manner, regardless of their p53 status. Cells treated with the MeOH extract exhibited morphological changes typical of apoptosis, such as rounding, shrinking, membrane blebbing, and detachment. Further fractionation of the extract led to the isolation of several compounds, including “(9*E*)-8-oxo-9-octadecenoic acid (1), (10*E*)-9-oxo-10-octadecenoic acid (2), (9*E*)-8-oxo-9-octadecenoate methyl ester (3), (9*Z*)-9-octadecenoate-(2′*S*)-2′,3′-dihydroxypropyl ester (4), (9*Z*)-9-octadecenoic acid (5), and palmitic acid (6)”. Among these, compounds 1 and 2 exhibited the strongest cytotoxic effects. The apoptosis induced by these compounds was associated with caspase-3 activation. These results highlight *A. spissacea* as a promising source for developing new anticancer agents [14].

### 3.4. Cordycipitaceae

#### 3.4.1. *Cordyceps militaris* 

A study by [20] examined the capacity of the water extract (WECM) of *Cordyceps militaris* (*C. militaris*) to induce apoptosis in human lung cancer A549 cells as well as how it influenced telomerase activity. The results revealed that the induction of Fas, catalytic activation of caspase 8, and Bid cleavage were linked to the growth inhibition and apoptotic induction caused by WECM treatment in A549 cells. In WECM-treated cells, caspases were also activated, anti-apoptotic Bcl-2 expression was downregulated, and pro-apoptotic Bax protein was upregulated. Furthermore, by the downregulation of c-myc, Sp1, and human telomerase reverse transcriptase (hTERT), WECM exhibited a dose-dependent suppression of telomerase activity. The collective findings of the research suggest that WECM promotes A549 cell apoptosis via intrinsic caspase pathways mediated by the mitochondria and extrinsic cascade of death receptors. Additionally, it was determined that, in evaluating the anti-tumor efficacy of *C. militaris* in human lung cancer cells, apoptotic events caused by WECM were linked with decreased telomerase activity through the inhibition of hTERT transcriptional activity [20].

#### 3.4.2. *Cordyceps taii* 

The folk remedy *Cordyceps taii* (*C. taii*) is indigenous to southern China and is believed to exhibit immunomodulatory, anticancer, and antimutagenic properties. In order to substantiate the ethnopharmacological claim regarding cancer prevention, in vitro and in vivo studies were conducted to examine the anticancer and antimetastatic properties of *C. taii* chloroform extract (CFCT). The chloroform extracts of *C. taii* were found to have antiproliferative activity against A549 lung cancer cells (in vitro). The chloroform extract exerted potent cytotoxic activity in a dose-dependent manner after 48 h of exposure. Furthermore, it was discovered that in tumor-bearing Kunming mice, CFCT greatly inhibited the growth of the tumor. Additionally, it raised the mice’s thymus and spleen indices. Histological analysis also illustrates that CFCT could induce tumor tissue necrosis. The possible mechanism of the anticancer and antimetastatic properties of CFCT may be related to anti-angiogenicity and activation of apoptosis [21].

### 3.5. Ganodermataceae

#### *Ganoderma lucidum* 

*Ganoderma lucidum* (*G. lucidum*) is a mushroom recognized by traditional Chinese medicine and commonly used in the forms of tea, powder, and dietary supplements [40]. Furthermore, 2.5% of the antlered form of *G. lucidum* in the diet reduced the quantity of lung cancer cells metastasis [41]. In a study by [24], “Water soluble polysaccharides isolated from *Ganoderma lucidum* (WSG) effectively suppressed cell viability and motility via inhibition of MAPK/ERK, PI3K/AKT, Smad and FAK signaling in lung cancer cells”. WSG was shown to limit the phosphorylation of intracellular molecules by facilitating the degradation of membrane growth factor receptors. This mechanism differs from the usual direct effect observed with small molecule inhibitors. This prevented the growth and proliferation of tumors. Furthermore, it was shown that ERK was an essential regulator of the TGFβR and EGFR-mediated signaling pathways and that it was critical for lung cancer cells. WSG simultaneously downregulated EGFR and TGFβR, which critically decreased ERK phosphorylation, even when mutated RAS was persistently activating its downstream signaling. The protein breakdown pathway and changes in the protein composition of lung tumor caused by WSG suggest that activation of the protein degradation system may be essential for a successful therapy of lung cancer with WSG. The study suggested that WSG’s inhibition of intracellular signaling could rely on the downregulation of membrane receptors. Furthermore, WSG was discovered to reduce EGFR and TGFβR protein levels during the short-term course of treatment. Remarkably, it was discovered that MG132, a proteasome inhibitor, prevented the WSG-induced decrease in EGFR and TGFβR protein levels. This implies that “the WSG regulation of EGFR and TGFβR proteins” involved a proteasome-mediated degradation process. Additionally, WSG could decrease other membrane proteins, like integrin, “enothelin receptor, and mesenchymal-like molecules”, indicating that WSG may have prevented the progression of lung tumors by focusing on various pathways. The research findings suggest that WSG efficiently suppressed lung cancer cells by disrupting the communication between Transforming Growth Factor β Receptor and the subsequent signaling cascades regulated by EGFR. More precisely, the suppression of cell growth and motility in lung cancer cells by WSG is attributed to the degradation of TGFβR and EGFR produced by WSG. WSG was found to substantially enhance the survival rate and inhibit the proliferation and metastasis of tumors in mice carrying LLC1 tumors, as observed in a mice tumor model. The findings indicated that WSG could serve as a potential candidate for suppressing lung cancer. Subsequent recommendations said that preclinical research is required to assess the safety and effectiveness of WSG when administered either alone or in conjunction with therapeutic medications to patients with lung cancer [24].

### 3.6. Hericiaceae

#### *Hericium erinaceus* 

One edible mushroom species with possible health benefits is *Hericium erinaceus* (*H. erinaceus*), frequently referred to as the “lion’s mane mushroom” in English. Studies on this specific mushroom’s main and secondary metabolites as well as its possible medical uses have been conducted [10]. In a study by [26], *H. erinaceus* mushroom peptides were isolated for their biological activity, and experiments were conducted to evaluate their potential against the lung cancer-simulating Chago-K1 cell line. The F4 sub-fraction showed antiproliferative influence against Chago-K1 and no cytotoxicity toward the normal human fibroblast cell line. Additionally, the fraction was tested for induction of apoptosis, and the results demonstrated growing percentages of early apoptotic cells at 24 h. The activity was better than that of the positive control (Doxorubicin). Chago-K1 cells were subjected to the F4 sub-fraction for 24 and 48 h in order to test for caspases 8 and 9. The F4 sub-fraction was found to be able to boost the activity of caspases 3, 8, and 9 as treatment duration increased the largest effects appeared at 48 h. The findings address both the intrinsic and extrinsic pathways of apoptosis by showing that the activation of caspases 3, 8, and 9 by the F4 sub-fraction was sufficient to induce apoptosis.

### 3.7. Hymenochaetaceae

#### *Phellinus linteus* 

In a study by [42], two bioactive extracts of *Phellinus linteus* (PL) demonstrated their cytotoxic effects on human lung cancer cell (A549) in vitro. It is likely that oxidative stress played a role in mediating this anticancer effect, ultimately leading to apoptosis. In the study by [33], PL has also been demonstrated to reduce tumor proliferation against human lung cancer H5800 lung epithelial LA4 cells. It was discovered that in lung cancer cells from mice and humans, PL mediated the following two functions: cell cycle arrest at a low concentration of PL and apoptosis in response to a high dose of PL. After exposure to a low dose of PL, G1 growth arrest occurred in the lung cancer cells. The decline in the activity of cyclin-dependent kinases CDK2, 4, and 6 is indicative of the negative growth control mediated by PL. However, PL caused dose-dependent apoptosis in lung cancer cells at high dosages. This was evidenced by DNA fragmentation, caspase activation, and loss of clonogenicity in the lung cancer cells, they were all absent from lung cancer cells treated with low doses of PL. Also, the normal mouse lung epithelial cells were subjected to low or high concentrations of PL. Complete suppression of PL-induced apoptosis was achieved by adding the caspase inhibitor Z-VADfmk. Furthermore, the low dosage of PL was able to complement doxorubicin in causing the lung cancer cells to undergo apoptosis.

### 3.8. Lyophyllaceae

#### *Termitomyces clypeatus* 

*Termitomyces clypeatus* is an edible mushroom that has ethnomedicinal uses [43]. In a study by [36], the aqueous extract of the mycelium cultivated in glucose medium contained main components identified by HPLC analysis as raffinose, sorbitol, and ribitol. It appeared that sorbitol and ribitol may be the byproduct of glucose utilization, whereas arabitol, inositol, and xylitol represent storage carbohydrates. The cytotoxicity of the extract was tested against A549 cells and was found to have a strong reduction in cell viability in a dose-dependent manner. In addition, treated mice showed a significant reduction in packed cell volume and tumor volume when compared to the control group. Mice treated with different concentrations of aqueous extract showed better hemoglobin content, RBC count, mean survival time, tumor inhibition, and percentage increase in life span in addition to significant reductions in tumor volume and viable tumor cell count. Thus, *Termitomyces clypeatus* possesses anticancer activity, which is valuable for application in food and drug products [36].

### 3.9. Meripilaceae

#### *Grifola frondosa* 

The Polyporaceae family’s maitake mushroom, or *Griffola frondosa*, is a well-known large mushroom that originated in northern Japan [44]. Maitake mushrooms are rich in several bioactive chemicals that contribute to their health advantages. These substances include polysaccharides, β-D-glucans, ergosterol, lactulose, dextrin, oligofructose, triterpenes, and various phenolic compounds [45,46]. More significantly, maitake produces a number of polysaccharide components that have antiviral, anti-inflammatory, anticancer, and immunomodulatory qualities [47,48,49]. In animal experiments, polysaccharides consisting of a ƒÀ-1,3 glucopyranoside main chain with ƒÀ-1,6-linked glucose branches b-glucan, termed D-Fraction, was isolated from the fruit body of the maitake mushroom. It has been demonstrated that D-Fraction increases the activity of immunocompetent cells, like NK cells, helper T cells, cytotoxic T cells, and macrophages, which reduces the size of tumors in mice without having any negative side effects [25,50,51]. Ten patients with stage II–IV lung cancer were given D-fraction therapy in human research, and the number of CD41 and CD81 cells, NK cell activity, and serum levels of sIL-2R were then tracked. According to the findings, D-Fraction was able to sustain and increase peripheral blood NK cell activity in lung cancer patients while also reversing the decline in T cell number and activity associated with malignancy [25]. In all patients analyzed, maitake D-fraction was found to reduce the expression of tumor markers, prevent the spread of metastatic disease, and boost NK cell function. Therefore, it seems that maitake D-Fraction inhibits the growth of cancer and mainly does so by activating NK cells. [25].

### 3.10. Omphalotaceae

#### *Lentinula edodes* 

The entire RNA of *Lentinula edodes* (*L. edodes*) was isolated, and a cDNA library was developed in order to assess the anti-tumor protein in the species. All of the transcriptome data for *L. edodes* was acquired. Latcripin-1 was identified as having an apoptosis-related role in the Cluster of Orthologous Groups of proteins database. The anticancer effects of Latcripin-1 on A549 cells were examined. After a 24-h incubation period, it was discovered that latcripin-1 pure proteins strongly inhibited the growth of A549 lung cancer cells, with the purified protein group showing a greater inhibition than the control group. Furthermore, protein Latcripin-1’s inhibitory effect on A549 cells was concentration- and time-dependent, with its inhibition level at various concentrations being higher than that of the positive control Cisplatin. Transmission electron microscopy showed distinct morphological changes in A549 cells after 24 h of treatment with Latcripin-1. In comparison, A549 cells in the control group retained their typical morphology, including a large nucleus, abundant mitochondria, and preserved membrane structure. Conversely, A549 cells treated with Latcripin-1 displayed increased intracytoplasmic vacuoles, chromatin condensation, nuclear fragmentation, and mitochondrial enlargement. In conclusion, the novel apoptosis-related protein Latcripin-1 from *L. edodes* was shown to induce apoptosis in A549 human lung cancer cells. The study was the first to identify and investigate the new protein Latcripin-1. However, more investigation is needed to clarify the apoptotic process it induces. The work lays the groundwork for future *L. edodes* anti-tumor research by providing fresh perspectives and benefits in the search for anti-tumor proteins as well as potential solutions to screening or purifying challenges [27].

### 3.11. Pleurotaceae

#### *Pleurotus eryngii* 

*Pleurotus eryngii* var. *ferulae* (*P. eryngii*, commonly known as Ferula mushroom) is a type of mushroom that usually grows in the dried roots of the poisonous Ferula plant. *P. eryngii* Quel protein (PEQP) was extracted from the dried fruiting bodies of *P. eryngii*, purified and characterized. It was then evaluated for in vitro antiproliferative activity in human non-small cell lung cancer A549. The results indicated that *P. eryngii* protein was less dangerous to normal cells (Chang cells) and more poisonous to tumor cells and also triggered the macrophage-mediated immune responses. Cytotoxicity results showed dose-dependent PEQP toxicity and presence of different organelle targets in tumor cells. Photomicrographs displayed that when PEQP concentration increased, tumor cells shrank, vacuoled, blebbing in the plasma membrane, detached gradually, and had condensation of chromatin and cytoplasm. However, cytotoxicity results suggested that PEQP may have more than one potential target in tumor cells. Therefore, *P. eryngii* protein has a potential use in functional foods as an active anti-tumor agent with immunomodulatory capabilities. However, more research is required to determine the exact mechanism by which PEQP produces these advantageous effects and whether it is clinically effective in cancer therapy [34].

### 3.12. Polyporaceae

#### 3.12.1. *Antrodia cinnamomea* 

*Antrodia cinnamomea* (*A. cinnamomea*) has long been used as a medicinal food to treat liver cancer, itchy skin, inflammation, and hypertension. [15]. According to some research, extracts from *A. cinnamomea*’s fruiting bodies and mycelia may have therapeutic promise as a chemotherapeutic agent against lung, prostate, breast, and hepatoma cells [52,53]. In a study by [15], *A. cinnamonomea*’s antimetastatic effects on highly metastatic CL1-5 cell lines were examined, along with the underlying mechanisms. The highly metastatic CL1-5 cells’ migration and motility were found to be inhibited by the ethanol extract of *A. cinnamomea* fruiting bodies in a concentration-dependent manner without any cytotoxicity. Gelatin zymography assay results showed that *A. cinnamonomea* reduced the activity of matrix metalloproteinases (MMP-2 and MMP-9) in a dose-dependent manner. Western blot analysis demonstrated that treatment with *A*. *cinnamonomea* lowered the expression of MMP-2 and MMP-9, while increasing the levels of their endogenous inhibitors, the tissue inhibitors of MMPs. More research showed that *A. cinnamomea* inhibited p38, JNK1/2, and ERK1/2 phosphorylation. Moreover, PI3K expression and Akt phosphorylation were inhibited by *A. cinnamonomea*. Additionally, the levels of “MMP-2 and MMP-9” were reduced in CL1-5 cells treated with specific inhibitors targeting PI3K, ERK1/2, JNK, and p38 MAPK. This research was the first to establish the anti-migration properties of this promising mushroom against human lung adenocarcinoma CL1-5 cells. To better understand the potential of ethanol extract as an anticancer drug, more preclinical and clinical research is needed.

#### 3.12.2. *Coriolus versicolor* 

Studies have shown that the turkey tail mushroom, also known as the cloud mushroom, *Trametes versicolor*/*Coriolus versicolor*, has anti-tumor properties against a variety of carcinomas [54]. Polysaccharide K, also known as PSK or Krestin, is isloated from the *C. versicolor* mushroom. In PSK, polysaccharide β-d-glucan chains are linked to a polypeptide moiety, making up roughly 38% of the molecule’s protein and 62% of its polysaccharide content. [55]. For almost thirty years, PSK, a protein-bound polysaccharide or proteoglycan, has been utilized as an adjuvant immunotherapy for various cancer types, including lung cancer [55]. Despite being initially isolated in 1971, PSK’s impact on lung cancer in particular has not been thoroughly examined [22]. In another study PSK significantly reduced proliferation of A549 lung cancer cells up to 80% [23]. Cell cycle arrest was generated, and when paired with IL-2, it boosted peripheral blood lymphocyte proliferation to 4.5-fold above control and raised the percentage of apoptotic cells by upregulating caspase 3 expression. According to Fritz et al. (2015) [22], in addition to directly inhibiting tumor growth, PSK stimulates several immune pathways that lead to the inhibition of cancer cells. These pathways include enhancing T-cell cytokine secretion, stimulating bone marrow and thymus activity, B-cell production of anti-tumor antibodies, boosting NK cell and cytotoxic T-cell activity, and activating macrophages and increasing tissue macrophage presentation of tumor antigen.

#### 3.12.3. *Lentinus squarrosulus* 

*Lentinus squarrosulus* (*L. squarrosulus*) is an edible mushroom commonly found in the wild. It is a white rot saprophytic fungus that thrives in forests on dead or decaying wood. Mushroom mycelia have been reported to be a good source of anticancer effects [56]. In a study by [28], following a six-hour treatment with peptide extracts, H460 lung cancer cells showed a dose-dependent cytotoxicity with a decline in cell viability. Treatment of H460 cells with peptide extracts at 40 µg/mL for 24 h led to an 85% decrease in viable cells. At the highest concentration tested, the peptide extracts caused both apoptosis and necrosis, as evidenced by DNA condensation with Hoechst 33342 and red fluorescence with propidium iodide (PI). To confirm the induction of apoptosis, Western blot analysis was used to evaluate apoptotic markers. There was an increase in cleaved-caspase-3, indicating activation of caspase-3, in H460 cells treated with the peptide extracts for 24 h. Additionally, PARP (Poly (ADP-ribose) polymerase-1) levels were significantly reduced, while cleaved-PARP levels increased in cells treated with 10–20 µg/mL of the extracts. The results suggest that the peptides from *L. squarrosulus* mushroom fruiting bodies primarily triggered apoptosis through the intrinsic mitochondrial pathway by decreasing Bcl-2 and increasing BAX. The extracts also reduced c-FLIP, an inhibitor of the death receptor-activated caspase cascade, which is associated with increased cleaved-caspase-8. Overall, the study demonstrates that peptides from *L. squarrosulus* mushrooms effectively induce apoptosis in lung cancer cells by decreasing anti-apoptotic Bcl-2 and c-FLIP proteins and increasing the pro-apoptotic protein Bax.

#### 3.12.4. *Lignosus rhinocerus* 

One of the most important medicinal mushrooms used by indigenous people in Southeast Asia and China is *Lignosus rhinocerus* (*L. rhinocerus*), also known as the tiger milk mushroom. The sclerotia of the cultured *L. rhinoceros* (via cold water extract) were found to be non-toxic to the comparable normal NL 20 human lung cells but to be cytotoxic to human lung carcinoma (A549). The mechanism of cell death induced by the cold-water extract of *L. rhinocerus* was studied using DNA fragmentation assays. The fragments of the DNA ladder on the treated A549 cells show that apoptosis was the mechanism causing the cell death. DNA fragmentation studies indicated that the cytotoxic effects of the extract on cancer cells were mediated through apoptosis. However the exact apoptosis mechanism of action was not explained in the study [30].

#### 3.12.5. *Lignosus tigris* 

*Lignosus* species from the Polyporaceae family, including eight varieties (*L. dimiticus*, *L. ekombitii*, *L. goetzii*, *L. sacer*, *L. rhinocerus*, *L. hainanensis*, *L. tigris*, and *L. cameronensis*), have long been valued for their therapeutic properties. The cold-water extract of *Lignosus tigris* was shown to reduce the viability of A549 cells while being non-toxic to normal NL20 lung cells. This cytotoxicity in A549 cells was linked to the induction of apoptosis through both extrinsic and intrinsic pathways. The extract enhanced the expression of caspase-8 and -9 and the pro-apoptotic Bax protein, while decreasing the expression of the anti-apoptotic protein Bcl-2 [31].

#### 3.12.6. *Trametes gibbosa*/*hirsuta* 

There are approximately 60 known species of *Trametes*, only a small number of which have been investigated for their potential therapeutic benefits, as records have shown that they have been used in traditional Chinese medicine to eliminate toxins, treat a variety of infections, strengthen, increase energy, support liver and spleen function, and strengthen immune response [38]. In the study by [38], 1H NMR spectroscopy analysis of the ethanol extracts showed that the mycelium extract was rich in metabolites, including triterpenes, sugars, polyphenols, and compounds from basidiocarps. The findings suggested that extracts from *Trametes* species have substantial medicinal potential, attributed to their diverse biologically active compounds and their synergistic effects. The extracts exhibited strong cytotoxic activity against human lung A549 cancer cell lines. When the extract’s cytotoxicity was tested on A549 cells, it was discovered that there was a dose-dependent decrease in cell viability. It was demonstrated through species comparisons that, in contrast to *Trametes gibbosa*, *Trametes hirsuta* had the highest potential for cytotoxicity [38].

### 3.13. Pseudoclitocybaceae

#### *Clitocybe alexandri* 

*Clitocybe alexandri* (*C. alexandri*) ethanol extracts’ ability to cause apoptosis in NCIH460 lung cancer cells was examined in a study by [18]. When the NCI-H460 cells were exposed to the ethanolic extract for 48 h, the proportion of apoptotic cells increased statistically considerably when compared to the blank cells. Additionally, the expression of few proteins that are related to the apoptotic process were also evaluated following treatment with mushroom extracts. The findings indicate that 48 h of treatment with the extract concentration in NCI-H460 cells resulted in an increase in wt p53, cleaved caspase-3, and cleaved poly ADP–ribose polymerase (PARP) levels. Following an HPLC-DAD analysis of the ethanolic extract, two phenolic acids and related compounds protocatechuic acid, p-hydroxybenzoic acid, and cinnamic acid were discovered and quantified. Cinnamic acid was found to be the most potent compound regarding cell growth inhibition. However, it was determined that the biggest reduction in viable cell count was achieved by using each drug concurrently. Overall, we discovered evidence of p53 and caspase-3-related changes in the cell cycle and apoptosis. The *C. alexandri* extract was found to raise the proportion of apoptotic cells and cause S-phase cell cycle arrest. Additionally, the findings indicates that the cytotoxicity induced by this mushroom extract is caused, at least in part, by the phenolic compounds found in the extract [18].

### 3.14. Suillaceae

#### *Suillus granulatus/luteus* 

*Suillus* species are related to pine trees and are primarily found in temperate regions of the Northern Hemisphere, while some have been brought to the Southern Hemisphere. The methanol extracts of *Suillus luteus*/*granulatus* were tested against Lewis lung carcinoma (3LL). Out of all the tested mushrooms the *Suillus* spp. (*granulatus* and *luteus*) exhibited a significant cytotoxic activity against the 3LL cells with *Suillus granulatus* being more active. *Suillus* spp. were found to contain cytotoxic compounds, such as the phenolic suillin, which was considered to be responsible for the in vitro cytotoxicity against Lewis lung carcinoma [35].

### 3.15. Thelephoraceae

#### *Thelephora ganbajun* 

*Thelephora ganbajun* (*T. ganbajun*) is becoming more well-known for its nutritional value and medicinal qualities, which include strong antioxidant, insect and bacterium resistance, and particular 5-lipoxygenase inhibitory effects, in addition to its well-liked flavor [37]. The antiproliferative activity of *T. ganbajun* was evaluated by [37]. The three extraction techniques used were water and ethanol, which are widely regarded as safe solvents, along with the ultrasound-assisted extraction (UAE) approach. In addition, rutin, 2-hydrocinnamic acid, and epicatechin were identified in the extract via the HPLC analysis. UAE demonstrated the most potent antiproliferative properties against A549 human lung cancer cells. The number of viable cell count was decreased, whereas the number of non-viable cell count was increased with treatment. In conclusion, *T. ganbajun* possessed significant antiproliferative activities toward human lung cancer [37].

### 3.16. Tricholomataceae

#### *Lepista inversa* 

The methanolic and ethanolic extracts of *Lepista inversa* (*L. inversa*) as well as the boiling water extract of its polysaccharides were assessed for their ability to suppress the growth of cancer cells in NCI-H460 lung cancer cells. The results revealed that *L. inversa* had the capacity to inhibit 50% of the growth in a human tumor cell line. Methanolic extracts of *L. inversa* proved to be generally more potent as compared to the polysaccharidic extract. This interesting growth inhibitory activity proves that this mushroom is a promising source of bioactive compounds [19]. Similar results were observed in a study by [29], where it was reported that methanolic extracts of fresh fruiting bodies of *L. inversa* had cytotoxic action against mouse tumor cell line 3LL (Lewis lung carcinoma).

### 3.17. Xylariaceae

#### *Xylaria hill* 

Ethyl acetate soluble (fraction E) of *Xylaria hill* (*X. hill*) fruiting body extract was evaluated for cytotoxicity A-549 by the MTT method. The findings showed that fraction E exhibited over 50% anticancer efficacy against A549 lung cancer cells. The preliminary chemical analysis of partially purified fraction E revealed the presence of active compounds, such as phenols, terpenoids, and polysaccharides. The study’s findings demonstrated that *X. hill* fruiting body extract exhibited efficient broad spectrum cytotoxic action against a human lung cancer cell line, which is due to the presence of phenols, terpenoids, and polysaccharides. Therefore, *X. hill* bioactive medicinal mushroom may be utilized as potential anticancer agents [39].

## 4. Discussion

The study yielded 26 mushrooms from 16 different families. The families ranged from Agaricaceae to Xylariaceae. The Polyporaceae family was the dominant family with seven mushrooms used to treat lung cancer. The mushroom extracts, fractions, compounds, and proteins were used to treat lung cancer. The extracts and fractions were prepared using various solvents, including methanol, ethanol, chloroform, hexane, ethyl acetate, and water. The solvents that were frequently used extraction were ethanol, water and methanol with ethanol being the predominantly used solvent. Over 10 species were extracted with ethanol, while over 8 were extracted with water, indicating that certain bioactive compounds may be non-polar, while others are semi-polar or polar. The three most evaluated cell lines were A549, NCI-H460, and 3LL. In this study, 65% of the mushrooms were evaluated using A549 adenocarcinoma, establishing it as the most extensively examined lung cancer cell line. This is logical, as lung adenocarcinoma is the predominant cause of lung cancer. Approximately 40% of lung tumors are adenocarcinomas, the most prevalent subtype, originating in the cells that lining the airway walls where mucus is generated [57].

**Figure 2:**
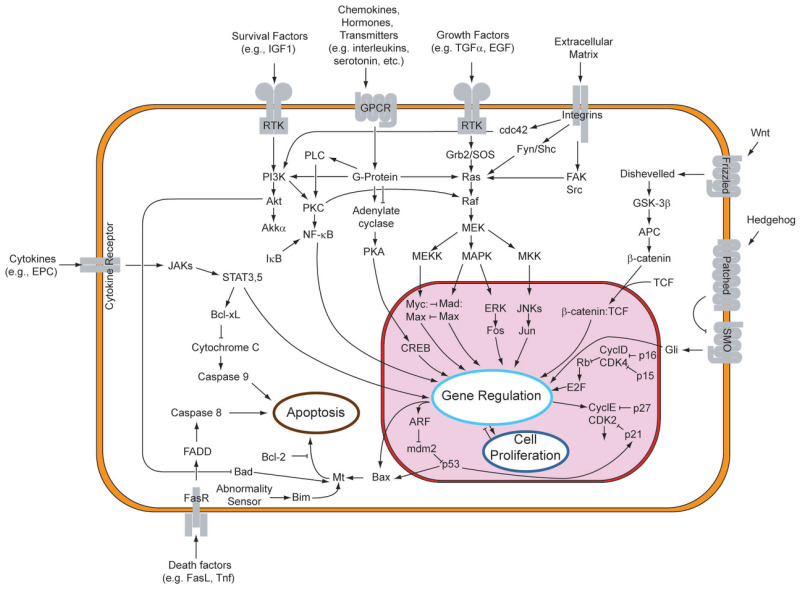
Overview of signal transduction pathways [58].

The primary mechanisms of actions include, the inhibition of cancer proliferation, cell cycle arrest, induction of cancer phagocytosis, suppression of tumor angiogenesis, immune system stimulation, and initiation of apoptotic cell death, facilitated by the regulation of caspases -3, -8, and -9, AKT, p27, p53, and the BAX/BCL2 ratio. The mentioned mechanism is governed by various signaling pathways indicated in Figure 2.

The studies cited were performed using in vitro (77%), in vivo (19%), and clinical trials (4%) experimental models. All mushroom species listed in Table 1 had anticancer potential and showed the ability to prevent cancer proliferation. The US National Cancer Institute Guidelines classify the cytotoxicity of extracts based on their in vitro antiproliferative activity against cancer cells. Extracts with an IC_50_ value below 20 μg/mL are deemed highly toxic; those with IC_50_ between 20 μg/mL and 50 μg/mL are classified as moderately toxic; extracts with IC_50_ from 50 μg/mL to 200 μg/mL are considered less toxic; and those with IC_50_ above 200 μg/mL are regarded as non-toxic [59]. The cytotoxicity of the mushrooms was evaluated using the MTT, XTT, and SRB colorimetric assays. All experiments were conducted a minimum of three times to ensure the credibility and reproducibility of the results. The four most toxic species against A549 cells were *X. hill* fraction (IC_50_ = 20 μg/mL, MTT), *L. edodes* Latcripin-1t protein (IC_50_ = 30 μg/mL, MTT), *C. taii* chloroform extract (IC_50_ = 30.2 μg/mL, SRB), and *T. clypeatus* water extracts (IC_50_ = 30 μg/mL, MTT). The least toxic species against A549 were *C. gigantea* methanol extract (IC_50_ > 500 μg/mL, XTT), *C. militaris* (IC_50_ > 500 μg/mL), and *L. rhinoceros* cold water extract (IC_50_ = 466.7 μg/mL). Despite the evaluation of the species through various assays under differing conditions, the findings reveal that three species (*X. hill*, *L. edode*, and *T. clypeatus*) among the top four were assessed using the MTT assay, allowing for a comparative conclusion that *X*. *hill* exhibited greater cytotoxicity activity relative to *L*. *edode* and *T*. *clypeatus*.

The *C. alexandri* and *L. inversa* mushrooms extracts were evaluated on NCI-H460 lung cancer cells using SRB assay. The results demonstrated that methanol, ethanol, and water extracts of *C. alexandri* were cytotoxic against NCI-H460 with IC_50_ of 34.8 μg/mL, 24.8 μg/mL, and 24.5 μg/mL, respectively. *L. inversa* methanol, ethanol, and water extracts showed a different picture with IC_50_ values of 36.3 μg/mL, 118.3 μg/mL, and 155.0 μg/mL. The findings demonstrated that the water and ethanol extracts of *C. alexandri* exhibited better efficacy compared to the water and ethanol extracts of *L. inversa* against NCI-H460. Comparable results were noted with the methanol extracts, where *C. alexandri* exhibited better activity compared to *L. inversa*. The aqueous extract of *C. alexandri* exhibited the highest toxicity against NCI-H460, with an IC_50_ of 24.5 μg/mL, followed closely by the ethanolic extract, which had an IC_50_ of 24.8 μg/mL. The investigations utilized the SRB assay under similar conditions for both species, allowing for the conclusion that *C. alexandri* exhibited better activity compared to *L. inversa* against NCI-H460 cells. Extracts of *S. granulatus* exhibited significant cytotoxicity against 3LL cells, with an IC_50_ of 6.8 µg/mL, surpassing the *S. luteus* extract, which had an IC_50_ of 40.3 µg/mL.

Some of the other species that showed anticancer potential were ethanol extracts from *M. procera*, which were tested for their effects on the proliferation of A549 cancer cells. The extract results demonstrated cytotoxic effects against A549 cells by inhibiting cell growth by >75% at the lowest concentration tested. *M. procera* exhibited a 94% inhibition of glucose 6-phosphate dehydrogenase in its anticancer mechanism against A549 cancer cells, which is essential for fulfilling the energy and metabolic requirements of glycolysis and the pentose phosphate pathway in cancer cells [32]. Methanol extracts of *A. spissacea* showed cytotoxicity against A549 cells. Following treatment with the methanol extract, the human lung cancer cells exhibited morphological alterations characteristic of apoptosis, including cell rounding, shrinkage, plasma membrane blebbing, and separation from the substratum. The cytotoxicity of the extracts was attributed to apoptosis, which was associated with the activation of caspase-3 [14].

Comparable outcomes were noted with the methanolic extracts of *A. lanipes* when evaluated on A549 cancer cells using the XTT assay. The results indicated an IC_50_ of <200 µg/mL after 72 h of incubation. Gene expression analysis indicated that the expression levels of Bcl-2 and CCND1 in A549 cells decreased in the dosage group compared to the control cells in a time-dependent manner. The expression of Bax, caspase-3, and p65 genes increased in the dosage group. The modification of these genes’ expression is believed to contribute to the sensitization of cancer cells to apoptosis [13]. The Western blot analysis of *C. gigantea* extract demonstrated a reduction in the expression of CDK4, Akt, and Bcl-2 proteins, whereas the expression of the pro-apoptotic protein Bax and the cell cycle checkpoint protein p53 rose following 72 h of incubation. The release of cytochrome c is linked to Bax and Bcl-2, leading to the activation of caspase-9 and caspase-3, which are closely connected to apoptosis. Procaspase-9 is activated by cytosolic cytochrome c, resulting in the activation of caspase-3, ultimately leading to apoptosis [17]. The extract induced apoptosis and cell cycle arrest by increasing the expression of caspase 3, caspase 9, BAX, and p53, while decreasing the levels of Akt, BCL2, CCND1, CCND2, and CDK4 [17].

**Figure 3:**
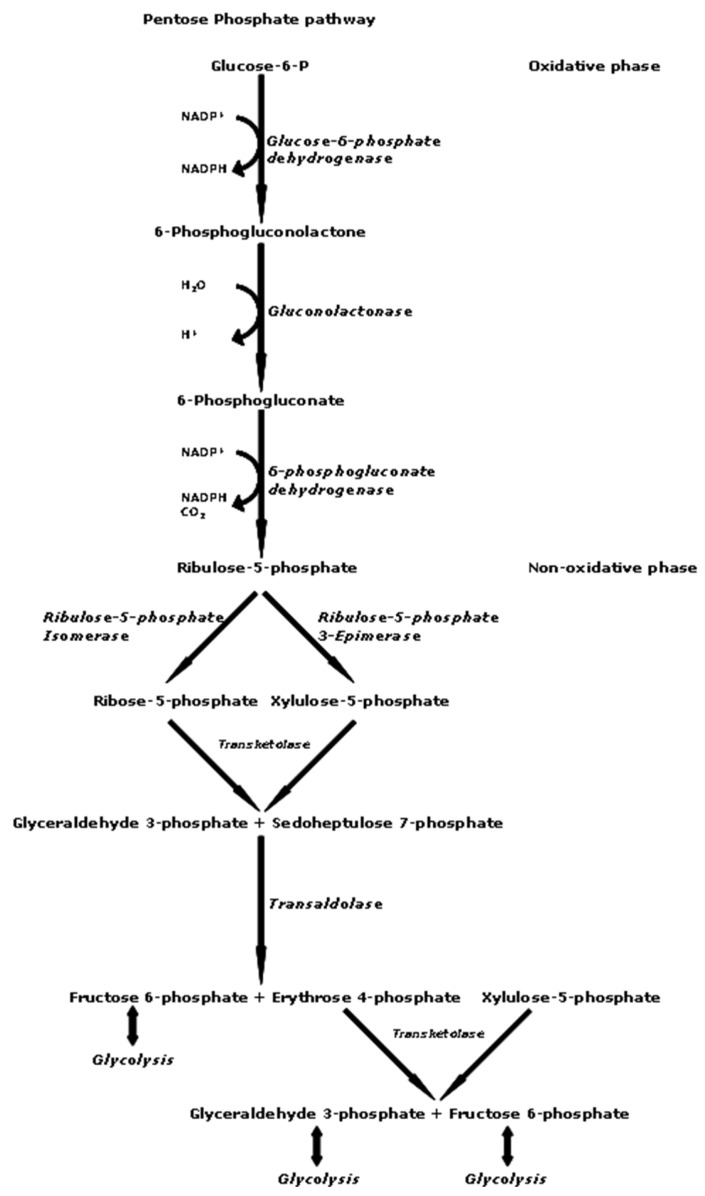
Pentose phosphate pathway [60].

Protein-bound polysaccharide, extracted from the CM-101 strain of the fungus *C. versicolor*, demonstrated in vitro suppression of tumor cell proliferation. The inhibition varied from 22% and 84%. Inhibition mechanisms were characterized by cell cycle arrest, resulting in cell accumulation in the G0/G1 phase, along with an increase in apoptosis and caspase-3 expression. The results demonstrate that PSK exhibits direct cytotoxic effects in vitro, impeding tumor cell proliferation [22]. The ethanol extracts of *A. cinnamonea* inhibited tumor growth and induced apoptosis via inhibiting the STAT3 signaling pathway. The extract reduced cellular motility, decreased the activity of matrix metalloproteinases two and nine, and increased the levels of tissue inhibitors of matrix metalloproteinases [16]. While ethanol extracts of *A. cinnamonea* utilized the STAT3 signaling pathway, the ethanol extracts of *M. procera* utilized the pentose phosphate pathway and demonstrated cytotoxic effects against A549 cells. *M. procera* inhibited G6PD as part of its anticancer mechanism against A549 lung cancer cells. Glucose 6-phosphate dehydrogenase and 6-phosphogluconate dehydrogenase from the pentose phosphate pathway (Figure 3) are crucial for providing the energy and biosynthetic demands of cancer cells through glycolysis and the pentose phosphate pathway [32].

Certain mushrooms, such as *P. linteus*, utilize alternative mechanisms, including oxidative stress induced by reactive oxygen species (ROS), to initiate apoptosis. The initiation of oxidative stress and its detrimental effects inhibit the proliferation of cancer cells. Likewise, the aqueous extract of *T. clypeatus* exhibited an anticancer effect that can be attributed to its antioxidant properties. The aqueous extract reduced lipid peroxidation and extended the lifespan of tumor-bearing mice. The aqueous extract reduced lipid peroxidation and extended the lifespan of tumor-bearing mice. ROS are essential to cellular signaling and the modulation of apoptosis pathways through the endoplasmic reticulum, mitochondria, and death receptors. ROS are generated by mitochondria and the metabolic functions of other organs. For certain normal functions of the cell, it is important to maintain a low content of ROS because they take part in the regulation of caspases during apoptosis [61].

The findings of this review demonstrate that a minimal number of in vivo studies have been conducted to date to evaluate the effects of mushrooms on lung cancer. The studies were done on mice models along with human trials. The chloroform extract of *C. taii* had an inhibitory effect on tumor growth in a murine model, resulting in decreased cell proliferation. Furthermore, it resulted in the death of tumor tissue and enhanced the activity of GSH-Px in several cancerous tissues [21].

D-Fraction, a β-glucan isolated using hot water and ethanol from the fruit body of the *G. frondosa* (maitake) mushroom, functions as a biological response modulator similar to lentinan found in *L. edodes* [25]. In animal experiments, D-fraction has been found to enhance the activity of immunocompetent cells, such as macrophages, helper T cells, cytotoxic T cells, and NK cells. In addition, orally administered D-fraction has been shown to reduce tumor size in mice without causing unwanted side effects [25]. D-Fraction has been shown in animal studies to increase the activity of immunocompetent cells, including macrophages, helper T cells, cytotoxic T cells, and NK cells. D-Fraction was also administered to lung cancer patients without anticancer drugs, and at the same time, NK cell activity was monitored to investigate whether the activity was closely related to disease progression. D-Fraction effectively increased the functionality of immunocompetent cells, including NK cells, helper T cells, cytotoxic T cells, and macrophages, without inducing any adverse side effects. The results suggest that D-fraction reverses the decrease in T cell number and activity seen in cancer and is capable of enhancing and maintaining peripheral blood NK cell activity in patients with lung cancer. The outcome is very interesting since the mushroom extract was able to inhibit cancer progression even without adjunct therapies [25]. However, combination therapies utilizing two or more anti-cancer medicinal products are commonly used due to their advantages for patients, enhancing both the safety and effectiveness of treatment. A combination of various mushroom species with polysaccharides (β-1,3-glucan or chitosan) or a mushroom extract containing vitamin C has demonstrated an enhancement in the anti-tumor efficacy of cancer therapy. A combination of mushroom extracts with chemotherapy also yields beneficial effects [61].

All the mushrooms that were examined in Table 1 showed the capacity to prevent lung cancer development through diverse modes of action. Most studies were performed using in vitro models; therefore, further research is necessary to examine the effects of mushrooms in in vivo models, given that they are ingested orally as traditional medicine. While oral administration of mushroom extracts is an inexpensive option for patients and may effectively stimulate the immune system to combat cancer, additional research on the limitations of these extracts is necessary. There are specific limitations with the conversion of extracts from preclinical to clinical applications, such as toxicity to normal cells. While most mushroom extracts listed in Table 1 were non-toxic to normal cells, the cold water extracts of *L. tigris* exhibited cytotoxicity against lung cancer A549 (IC_50_ of 70.67 µg/mL). However, it also showed cytotoxic effects on the non-tumorigenic lung NL20 (IC_50_ value of 71.83 µg/mL). Such results undermine the conventional application of the extract, as it is unstable for human intake due to its toxicity to normal cells. Moreover, investigating these in vitro cytotoxicity effects on the animal models will also add more insight into the safety of mushroom consumption, which is limited currently. Standardization of extracts (traditional remedies) is valuable in quality control and in ensuring that correct doses and sufficient active substances are present in the traditional remedies. Cultivation and sustainability of mushrooms as sources of active ingredients should also be considered when developing herbal remedies.

## 5. Conclusions

Mushrooms are a type of food that is highly regarded for its culinary qualities and several health benefits. Although they have been used for a very long time in many different cultures, they are currently supported by logical scientific research. Because medicinal mushrooms have an abundance of bioactive compounds, relatively few of which have been documented and many more of which are still being identified, they are becoming a more significant part of the pharmaceutical industry. This review demonstrated the potential use of mushrooms and their anticancer mechanisms in lung cancer treatment in vitro and in vivo. The current findings and information may offer fresh perspectives on the potential medicinal applications of mushrooms as well as valuable recommendations for the development of anti-tumor medications derived from mushrooms to treat lung cancer. Antiproliferative agents, such as mushrooms, that can induce selective cytotoxicity against cancer cells, without causing much harm to normal cells, are highly desirable for therapeutic purposes and may be considered in the development of novel cancer chemotherapeutic drugs. These cytotoxic abilities of mushroom products may potentially play a significant role in treating selected cancers by working in synergy with conventional chemotherapeutic drugs, thereby improving their efficacy or reducing their toxicity. The pharmacological effects of mushroom remedies warrant more investigation, particularly regarding the distribution of substances inside the body following intake. The remedies must be tested with in vivo models and clinical trials prior to consumption to comprehensively ascertain their underlying effects, including potential undesirable side effects. Clinical trials, in conjunction with in vivo models, are essential in determining a standard dose that is both effective and safe for oral administration. However, the cultivation of mushrooms must be carefully regulated during remedy preparation since excessive cultivation may result in the extinction of the species. In most of the studies, the positive controls, which are the chemotherapeutic drugs, such as Doxorubicin, Cisplatin, and Cyclophosphamide had better activity than the mushrooms extract however synergy with the mushrooms also increased their efficacy. Mushrooms may induce numerous side effects, including gastrointestinal distress, dizziness, nausea, and allergic reactions, contingent upon the kind of mushroom. Thus, more investigation is required to investigate the potential of medicinal mushrooms as a fundamental component of a balanced diet and lifestyle to prevent the development of cancer.

## Figures and Tables

**Table 1:** Anticancer mechanisms and phytochemicals of mushrooms used to treat lung cancer.

Species	Family	Experimental Model	Solvent/Fraction	Mechanism/Results	Reference
*Agaricus lanipes*	Agaricaceae	In vitro (A549 cells)	Methanol extract	Strong pro-apoptotic and antiproliferative effects were demonstrated by the extract.	[13]
*Amanita spissacea*	Amanitaceae	In vitro (A549, H1264, H1299 and Calu-6 cells cells)	Methanol extract	The cytotoxicity of the extracts was attributed to apoptosis, which was associated with the activation of caspase-3.	[14]
*Antrodia cinnamonea*	Polyporaceae	In vivo (LLC and CL1-5 cells) In vitro (CL1-0 cells)	Ethanol extract	The treatment inhibited the growth of the tumor and triggered programmed cell death by obstructing the STAT3 signaling pathway. The extract reduced cell movement and mobility, lowered the activity of matrix metalloproteinases two and nine, and increased the levels of tissue inhibitors of matrix metalloproteinases.	[15,16]
*Calvatia gigantea*	Agaricaceae	In vitro (A549 cells)	Methanol extract	The extract facilitated apoptosis and cell cycle arrest by enhancing the expression of caspase 3, caspase 9, BAX, and p53 and reducing the levels of Akt, BCL2, CCND1, CCND2, and CDK4.	[17]
*Clitocybe alexandri*	Pseudoclitocybaceae	In vitro (NCI-H460 cells)	Ethanol extract and hot water extracts	The extract halted cell progression at the S-phase, which led to a rise in apoptotic cells. “The fraction triggered apoptosis by linking the p53 and caspase-3 pathways” with cell proliferation during the cell cycle.	[18,19]
*Cordyceps militaris*	Cordycipitaceae	In vitro (A549 cells)	Water extract	The caspase pathway is mediated by mitochondria and the receptor-driven death signaling cascade. In addition, the extract inhibited the transcription of hTERT, which is activated by decreases in telomerase induced by apoptosis.	[20]
*Cordyceps taii*	Cordycipitaceae	In vitro (A549 cells)	Chloroform extract	The extract demonstrated an inhibitory effect on tumor growth in the mouse model, resulting in a decrease in cell proliferation. Moreover, it caused the death of tumor tissue and increased the activity of the GSH-Px in different cancer tissues.	[21]
*Coriolus versicolor*	Polyporaceae	In vitro (A549 cells)	Ethanol extract	The extract reduced the development of A549 cancer cells.	[22,23]
*Ganoderma lucidum*	Ganodermataceae	In vitro (3LL cells). “In vivo mouse model (MDA-MB-231 cells)”	“Ethanol extract with 6% triterpenes and 13.5% polysaccharides”.	The fraction exhibited anti-cancer properties by inhibiting cell proliferation. Orally administering the extract inhibited tumor growth and suppressed cancer cell growth.	[24]
*Grifola frondosa*	Meripilaceae	In vivo (human patients)	Water extract	The extract has been shown to enhance the function of immunocompetent cells, such as NK cells, helper T cells, cytotoxic T cells, and macrophages, without causing any adverse effects. This leads to a reduction in tumor diameter in mice.	[25]
*Hericium erinaceus*	Hericiaceae	In vitro Chago-K1 cells	Ethanol extract and peptides	Apoptosis was induced by the in vitro antiproliferative activity in human lung cancer cell lines (Chago-K1). Additionally, the expression of caspases 3, 8, and 9 in Chago-K1 cells increased following treatment.	[26]
*Lentinula edodes*	Marasmiaceae	In vitro (A549 cells)	Latcripin-1t protein	The protein had an inhibitory impact on the growth of cancerous cells.	[27]
*Lentinus squarrosulus*	Polyporaceae	In vitro H460 cells	Water extract	The extracts induced apoptosis in lung tumor cells by reducing the levels of anti-apoptotic proteins Bcl-2 and c-FLIP, while increasing the levels of the pro-apoptotic protein Bax.	[28]
*Lepista inversa*	Tricholomataceae	In vitro (NCI-H460 cells)	The extracts obtained are phenolic, derived from methanol and ethanol, and polysaccharidic, obtained by heating water.	The fraction demonstrated efficacy in inhibiting the growth of lung cancer cells.	[19,29]
*Lignosus rhinocerus*	Polyporaceae	In vitro (A549 cells)	Cold water extract derived from sclerotia	The extract showed activity in reducing cancer cell proliferation.	[30]
*Lignosus tigris*	Polyporaceae	“In vivo—mice model and in vitro (A549 cells)”	Cold water extract of sclerotia	Through both intrinsic and extrinsic signaling mechanisms, the extract induced cellular apoptosis and had tumor growth suppressive impact. Proapoptotic proteins BAX, caspase-8, and -9 were all expressed in response to apoptosis, but BCL2 expression was suppressed concurrently.	[31]
*Macrolepiota procera*	Agaricaceae	In vitro (A549 cells)	Ethanol extract	The A549 cells’ viability was decreased by the extracts. It also inhibited the enzymatic activity of 6-phosphogluconate dehydrogenase (6PGD) and glucose 6 phosphate dehydrogenase (G6PD).	[32]
*Phellinus linteus*	Hymenochaetaceae	The study utilized a mice model with LKR cells and conducted in vitro experiments using H5800 and A549cells.	Ethanol extract	The onset of oxidative stress and its lethal impact prevented cancer cells from proliferating. Furthermore, because caspase-3 and -9 were expressed more frequently in the extract, it caused apoptotic cell death. The primary mechanisms by which the extract induced apoptosis was DNA fragmentation, proapoptotic caspase activation, and clonogenicity loss.	[33]
*Pleurotus eryngii*	Pleurotaceae	In vitro (A549)	Quel protein (PEQP)	The study showed that PEQP was cytotoxic to A549 non-small cell lung cancer cells, non-toxic to Chang cells, and able to activate macrophages.	[34]
*Suillus granulatus*	Suillaceae	In vitro (3LL cells)	Methanol extract	The fraction had inhibitory effects on the proliferation of cancer cells.	[35]
*Suillus luteus*	Suillaceae	In vitro (3LL cells)	Methanol extract	The extract exhibited anticancer cell proliferation inhibitory properties.	[35]
*Termitomyces clypeatus*	Lyophyllaceae	In vitro (A549 cells)	Water-soluble extract	The therapy demonstrated an anticancer impact that can be attributed to its antioxidant property. The aqueous extract decreased lipid peroxidation and increased the lifespan of mice with EAC tumors.	[36]
*Thelephora ganbajun*	Thelephoraceae	In vitro (A549 cells)	Ethanol extract	The extract exhibited anticancer cell proliferation inhibitory.	[37]
*Trametes gibbosa*	Polyporaceae	In vitro (A549 cells)	Ethanol extract	The therapy effectively hindered the growth of cancer cells.	[38]
*Trametes hirsuta*	Polyporaceae	In vitro (A549 cells)	Ethanol extract	The treatment showed antiproliferative efficacy on cancer cells.	[38]
*Xylaria Hill*	Xylariaceae	In vitro (A549 cells)	“Hexane, ethyl acetate, and methanol extracts”	The extracts exhibited an inhibitory effect on cell growth, and there were also changes in the morphology of A549 cells.	[39]

## Data Availability

The data used to support the findings of the study are available from the corresponding author upon request.
